# An anatomically correct 3D‐printed mouse phantom for magnetic particle imaging studies

**DOI:** 10.1002/btm2.10299

**Published:** 2022-03-01

**Authors:** Nicole S. Sarna, Leyda Marrero‐Morales, Ryan DeGroff, Angelie Rivera‐Rodriguez, Sitong Liu, Andreina Chiu‐Lam, Hayden J. Good, Carlos M. Rinaldi‐Ramos

**Affiliations:** ^1^ J. Crayton Pruitt Family Department of Biomedical Engineering University of Florida Gainesville Florida USA; ^2^ Department of Chemical Engineering University of Florida Gainesville Florida USA

**Keywords:** 3D printing, animal replacement, imaging phantom, magnetic particle imaging

## Abstract

We report anatomically correct 3D‐printed mouse phantoms that can be used to plan experiments and evaluate analysis protocols for magnetic particle imaging (MPI) studies. The 3D‐printed phantoms were based on the Digimouse 3D whole body mouse atlas and incorporate cavities representative of a liver, brain tumor, and orthotopic breast cancer tumor placed in anatomically correct locations, allowing evaluation of the effect of precise doses of MPI tracer. To illustrate their use, a constant tracer iron mass was present in the liver for the breast (200 μg_Fe_) and brain tumor (10 μg_Fe_) model, respectively, while a series of decreasing tracer iron mass was placed in the tumor region. MPI scans were acquired in 2D and 3D high sensitivity and high sensitivity/high resolution (HSHR) modes using a MOMENTUM imager. A thresholding algorithm was used to define regions of interest (ROIs) in the scans and the tracer mass in the liver and tumors was calculated by comparison of the signal in their respective ROI against that of known mass fiducials that were included in each scan. The results demonstrate that this approach to image analysis provides accurate estimates of tracer mass. Additionally, the results show how the limit of detection in MPI is sensitive to the details of tracer distribution in the subject, as we found that a greater tracer mass in the liver cavity resulted in poorer sensitivity in tumor regions. These experiments illustrate the utility of the reported 3D‐printed anatomically correct mouse phantoms in evaluating methods to analyze MPI scans and plan in vivo experiments.

## 1 INTRODUCTION

Magnetic particle imaging (MPI) is a new tomographic molecular imaging modality with increasing potential clinical application.^1^ With MPI, one can visualize and quantify the distribution of biocompatible superparamagnetic iron oxide nanoparticle (SPION) tracers. To achieve this, two opposing quasi‐static magnetic fields are applied to create a magnetic gradient field with a field‐free region (FFR) in the center, while a uniform alternating magnetic field (AMF) is applied to excite the nanoparticles.^2^ SPIONs in the FFR respond to the AMF, generating a signal detected by the MPI receive coils.^3^ An image is constructed by scanning the FFR over a field of view (FOV). MPI possesses many desirable characteristics for in vivo studies, such as negligible signal from anatomical features, complete tissue penetration, and MPI signal intensity that is proportional to SPION mass, which enables quantification.^1^ Since SPIONs can be used to label cells, this makes MPI ideal for cell tracking.^4^ Additionally, because SPIONs can be made to release heat that can be used to destroy cancer, MPI can be valuable in magnetic fluid hyperthermia (MFH)^2^ studies.

In a typical MPI study, a dose of SPION tracer is administered to a subject, followed by MPI scan acquisition at selected time points. Quantification of SPION distribution is carried out by comparing the tracer signal in regions of interest (ROI) to that of known mass fiducials. While simple in principle, there are many parameters and choices that can impact the sensitivity and accuracy of SPION quantification using MPI. Obvious factors include the choice of tracer and the MPI acquisition parameters of excitation field strength and frequency and gradient field magnitude. For the commercially available MOMENTUM™ imager from Magnetic Insight, the latter correspond to the choice of imaging mode, with four standard imaging modes available (default, high sensitivity [HS], high resolution [HR], and high sensitivity/high resolution [HSHR]) that vary according to the strength of the gradient field and other details of signal acquisition and processing. Other factors that significantly influence MPI quantification results are the preparation and positioning of known mass fiducials (whether in the same scan or in separate scans), the position and orientation of the subject, and the process of image analysis and processing to estimate tracer mass in a ROI. Most MPI studies to date have focused on proof‐of‐principle demonstrations of potential applications. However, as the field continues to mature, we expect that increasing attention will be needed to address the role of the above factors on the accuracy and sensitivity of MPI to quantify SPION distribution in living subjects. Because of the large number of factors at play, we hypothesize that MPI‐compatible phantoms that allow positioning of precise tracer masses in anatomically correct positions mimicking major organs that accumulate SPION tracers would be immensely valuable to advance the field of MPI.

Historically, phantoms have been used to simulate physiological phenomena, determine appropriate tracer dosages, optimize image quality, and evaluate a system's imaging capabilities.^5^ The creation of imaging phantoms has evolved due to recent advances with three‐dimensional (3D) printing, which has enabled rapid, low‐cost, and reproducible phantom development.^6^ 3D‐printed phantoms for MPI studies have been primarily utilized to evaluate instrument performance^7^, ^8^, ^9^ and SPION tracer characteristics^10^, ^11^ to ensure reproducibility of experiments in the field. In addition, phantoms have been used to explore potential biomedical applications of MPI such as magnetic hyperthermia,^12^ measurement of blood flow velocity,^13^ quantification of vascular stenoses,^14^ and stent placement guidance.^15^ However, the use of an anatomically correct animal phantom has not been reported for MPI studies. An anatomically correct phantom is desirable because it could mimic the results from in vivo studies and help answer important questions regarding the most suitable MPI data acquisition conditions, image analysis methods, and SPION dose experimental parameters. While MPI already contributes to advancing the ethical principles of replacement, reduction, and refinement (3Rs) as a noninvasive imaging modality, anatomically correct phantoms would further enable the planning and execution of MPI studies and image analysis workflows prior to animal experimentation.^16^

Multiple studies have presented the development of digital mouse atlases that model structures of live mouse anatomy that can be converted to solid parts to serve as a molecular imaging phantoms.^17^, ^18^, ^19^, ^20^, ^21^ These atlases differ in the specific organs that are modeled and the developmental stage of the mouse and are intended to assist in vivo studies for a variety of imaging systems. The Digimouse atlas, reported by Dogdas et al., is a whole‐body volumetric mouse atlas that has delineated organs of a normal nude male mouse, including the brain, heart, liver, lungs, stomach, and skin surface, among others.^17^ With the incorporation of numerous major organs in anatomically correct locations, phantoms created from the Digimouse atlas, such as the one reported here, can be highly versatile and designed to represent specific disease models.

In this article, we report two 3D‐printed mouse phantoms suitable for planning and evaluation of MPI studies. These phantoms were created using the Digimouse atlas and possess anatomically correct locations of liver cavities and breast and brain tumors. These were used to evaluate the effect of signal from nonspecific tracer accumulation in the liver on the detection and quantification of tracer signal in the tumor sites using a previously reported tracer tailored for MPI.^11^

## 2 RESULTS

**FIGURE 1:**
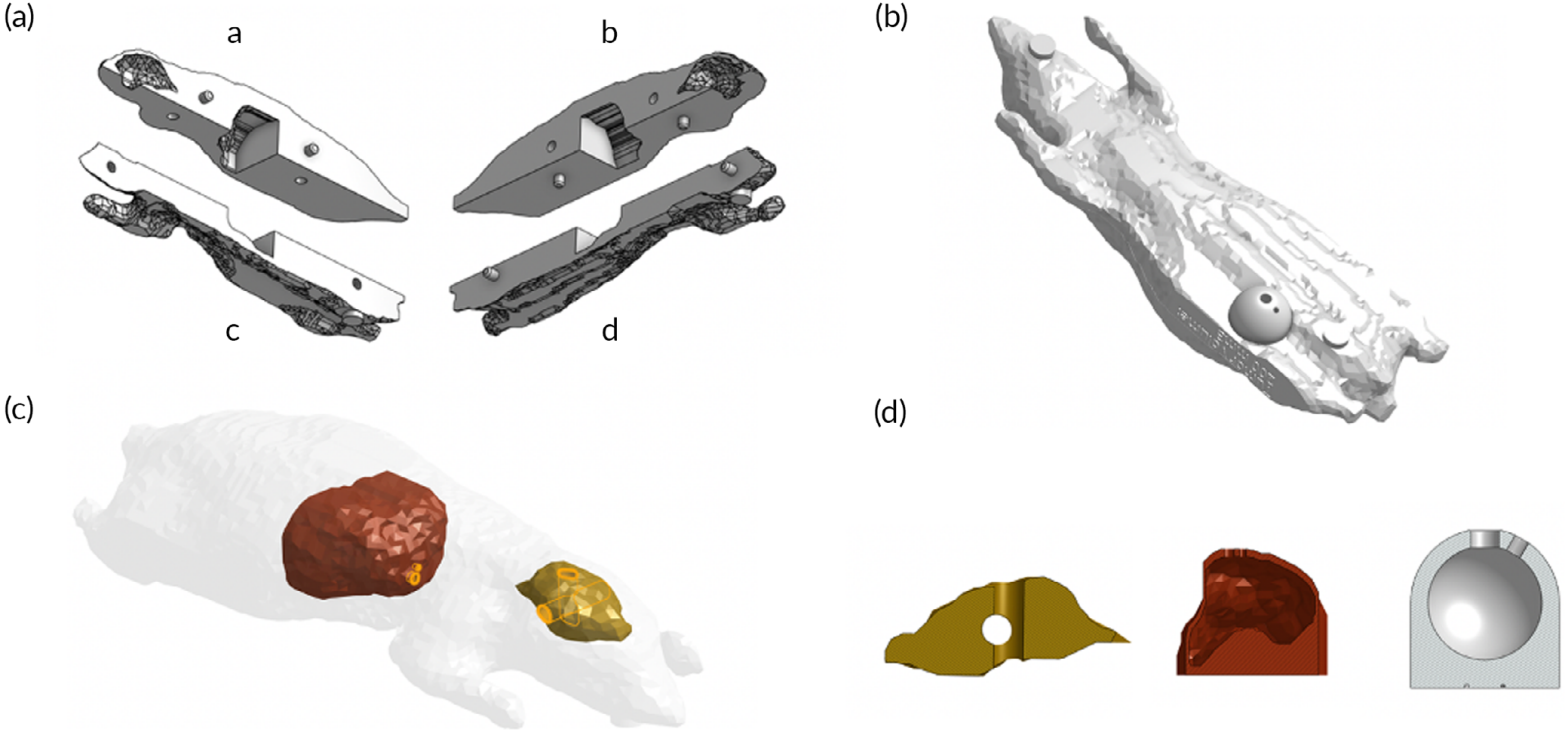
(a) Rendering of the four‐part mouse body assembly with cavities for the 3D‐printed mouse liver and brain. A transverse slice separates dorsal parts (A and B) from ventral parts (C and D); a sagittal slice separates lateral pairs (A and C; B and D). (b) Rendering of a 100 mm^3^ breast tumor model in wholemouse assembly. Part C was configured to fit breast tumor phantoms of various volumes. (c) Rendering of the liver (red) and brain (gold) inside the mouse. (d) Cross sections of the brain, liver, and breast tumor phantom

The 3D‐printed mouse phantom holds a hollow liver with a 450 mm^3^ filling capacity and a brain with two orthogonal cylindrical cavities for capillary tubes loaded with SPION tracer. A flat base was added to the liver for SPION loading convenience and does not contribute to the volume capacity. Due to inconsistencies when 3D printing the hollow liver, the maximum SPION volume loaded in the liver cavity was approximately 375 μL. The four‐part mouse body assembly secures the liver and brain in place and allows interchangeability so that a custom right flank for breast tumor studies may be added to the assembly, as illustrated in Figure 1. A configuration of hollow, spherical breast tumor phantoms with eight different volumes ranging from 10 to 1000 mm^3^ were created in Onshape®. For these studies, the 100 mm^3^ breast tumor cavity was used. Each tumor fits into the same 9.8 mm diameter cavity in the custom ventral right flank piece. Figure 1 outlines the geometry of the liver, brain, and breast tumor phantoms.

The brain and breast tumor phantoms contained a constant mass of iron in the liver cavity, while the tumor ROIs were loaded with a dilution series of RL‐1 tracer. Table 1 summarizes the iron mass present in the tumor and liver sites for both models. The iron content present in the liver in each model and dilution series was chosen to mimic relevant studies that implement SPION administration. For the brain tumor model, an iron mass of ~10 μg_Fe_ was chosen based on a previous study that used MPI to monitor localization of SPION labeled T cell in the brain following adoptive cell transfer (ACT).^4^ In that study, 10^7^ T cells loaded with approximately 1 pg_Fe_/cell were administered intravenously and we observed most of the signal in the liver, with a much smaller signal in the brain of mice bearing brain tumors. Thus, the liver mass and brain tumor dilutions were chosen to represent a situation where most of the administered tracer ends up in the liver and a small amount of tracer ends up in the brain tumor. In contrast, an iron mass of ~200 μg_Fe_ in the liver for the breast tumor model was chosen to mimic MFH studies, where much larger doses are administered to achieve a significant temperature rise in the tumor. In that case, a mass of ~200 μg_Fe_ in the liver could correspond to a case where a dose of 10 mgFe/kg is administered to a 20 g mouse intravenously and most of the tracer accumulates in the liver.

**TABLE 1:** Dilution series tracer mass for brain tumor and breast tumor phantom studies. Total iron mass was calculated by the 1,10‐phenanthroline colorimetric assay for iron quantification

		Brain tumor model	Breast tumor model
Cavity	Dilution series	Total iron mass (μg_Fe_) in 1 μL	Total iron mass (μg_Fe_) in 100 μL
Tumor	1	2.05	205.0
	2	1.025	102.5
	3	0.513	51.25
	4	0.256	25.63
	5	0.128	12.81
	6	0.064	6.41
	7	0.032	3.20
	8	0.016	1.60
	9	0.008	0.8
Liver		Total iron mass (μg_Fe_) in 375 μL	Total iron mass (μg_Fe_) in 375 μL
		10.3	205

**FIGURE 2:**
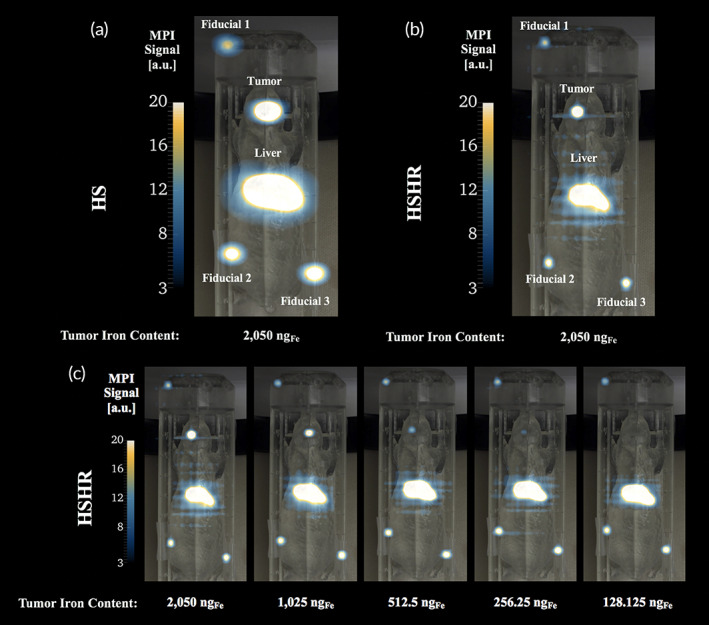
Dilution series in the brain tumor phantom study in 2D high‐sensitivity (HS) and 2D high‐sensitivity/high‐resolution (HSHR) scanning modes. Representative image of the (a) 2D HS and (b) 2D HSHR scanning modes. (c) Montage of images for the dilution series in HSHR mode in the brain tumor model demonstrating a signal decrease in the tumor region of interest (ROI). The color lookup table range was kept the same for all magnetic particle imaging (MPI) scans and was selected to best visualize the brain tumor signal at low concentrations. Fiducial 1, 2, and 3 contain an iron mass of 0.5_,_ 1, and 1 μg_Fe_, respectively, and the liver contains 10.3 μg_Fe_

**FIGURE 3:**
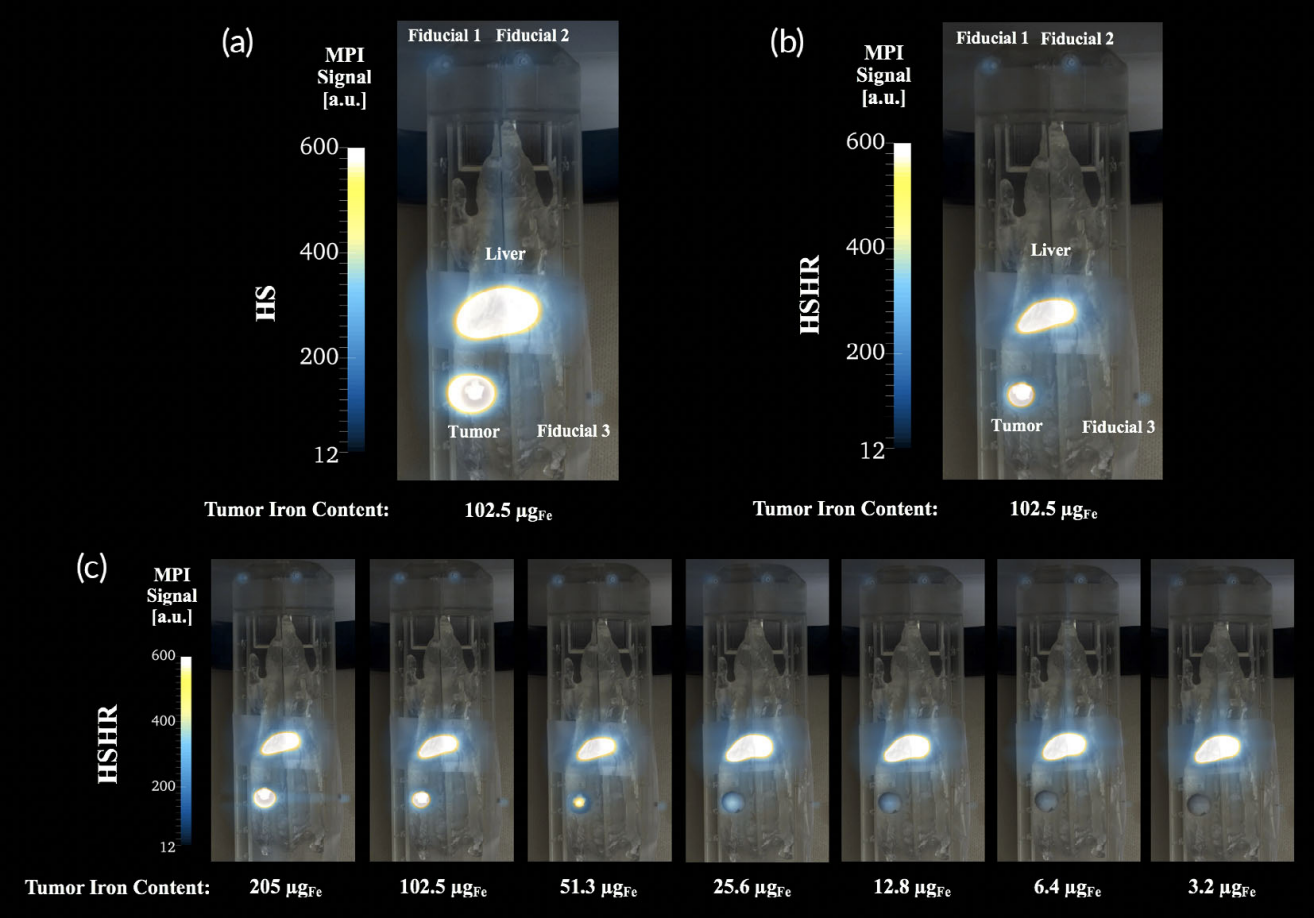
Dilution series in the breast tumor phantom study in 2D high‐sensitivity (HS) and 2D high‐sensitivity/high‐resolution (HSHR) scanning modes. Representative image of the (a) 2D HS and (b) HSHR scanning mode. (c) Montage of images for the dilution series in HSHR mode in the breast tumor model demonstrating a signal decrease in the tumor region of interest (ROI). The color lookup table range was kept the same for all magnetic particle imaging (MPI) scans and was selected to best visualize the breast tumor signal at low concentrations. The color range may make it difficult to visualize fiducials. Fiducial 1, 2, and 3, contain an iron mass of 2, 2, and 1 μg_Fe_, respectively, and the liver contains 205 μg_Fe_

Representative results, consisting of MPI images overlayed with optical images of the phantoms are presented in Figure 2 for the brain tumor model and in Figure 3 for the breast tumor model. We note that the color scale ranges were chosen in Figures 2 and 3 to accentuate the signal from the tumor region in the lowest mass dilution that was visible, thus oversaturating the liver and fiducials in all images. Furthermore, we note that we assume that a signal with a mean signal‐to‐noise‐ratio (mSNR) greater than 3 indicates a signal that is statistically different from the background^22^ and therefore above the limit of detection (LoD).

For the brain tumor model (Figure 2), the lowest tracer mass above the LoD corresponded to 256.25 ng_Fe_ in 2D HS (mSNR = 4.3) and 2D HSHR (mSNR = 5.1) scanning modes, with approximately 10 μg_Fe_ present in the liver. MPI scans were coregistered with the optical images of the loaded 3D‐printed phantom for HS (Figure 2a) and HSHR (Figure 2b) scanning modes (shown for brain tumor loaded with 2.05 μg_Fe_). A decrease in iron mass in the tumor corresponded to a decrease in signal intensity in the tumor ROI, shown as an example for the HSHR dilution series in Figure 2c. A montage of the HS dilution series is shown in Figure S1 in the Supporting Information. Upon inspection of the MPI scans, the signal for the brain tumor ROI with 128.1 ng_Fe_ was below the LoD for both the 2D HS (mSNR = 2.0) and 2D HSHR (mSNR = 1.9) scanning modes. Additionally, in the 3D HS scan mode, the signal for the brain tumor ROI with 128.1 ng_Fe_ was above the LoD (mSNR = 3.8), while the signal for the brain tumor ROI with 64.1 ng_Fe_ was below the LoD (mSNR = 0.8). A representative 3D HS MPI scan coregistered with a CT scan of the mouse phantom is shown in Movie S1, with 2.05 μg_Fe_ in the brain tumor ROI.

For the breast tumor model (Figure 3), the lowest tracer mass with a signal above the LoD corresponded to 6.41 μg_Fe_ in 2D HS (mSNR = 3.4) and 2D HSHR (mSNR = 4.5) scanning modes, with the presence of approximately 200 μg_Fe_ in the liver. MPI scans were coregistered with the optical images of the loaded 3D‐printed phantom for HS (Figure 3a) and HSHR (Figure 3b) scanning modes (shown for brain tumor loaded with 102.5 μg_Fe_). A decrease in iron mass in the tumor corresponded to a decrease in signal intensity in the tumor ROI, shown as an example for the HSHR dilution series in Figure 3c. A montage of the HS dilution series is shown in Figure S2 in the Supporting Information. Upon inspection of the MPI scans, the signal for the breast tumor ROI with 3.2 μg_Fe_ is below the LoD for both the 2D HS (mSNR = 2.2) and 2D HSHR (mSNR = 2.8) scanning modes. Additionally, in the 3D HSHR scan mode, the signal for the breast tumor ROI with 1.6 μg_Fe_ was above the LoD (mSNR = 6.4), but the signal for the breast tumor ROI with 0.8 μg_Fe_ was below the LoD (mSNR = 1.9). A representative 3D HS MPI scan coregistered with a CT scan of the mouse phantom is shown in Movie S2, with 102.5 μg_Fe_ in the breast tumor ROI.

**FIGURE 4:**
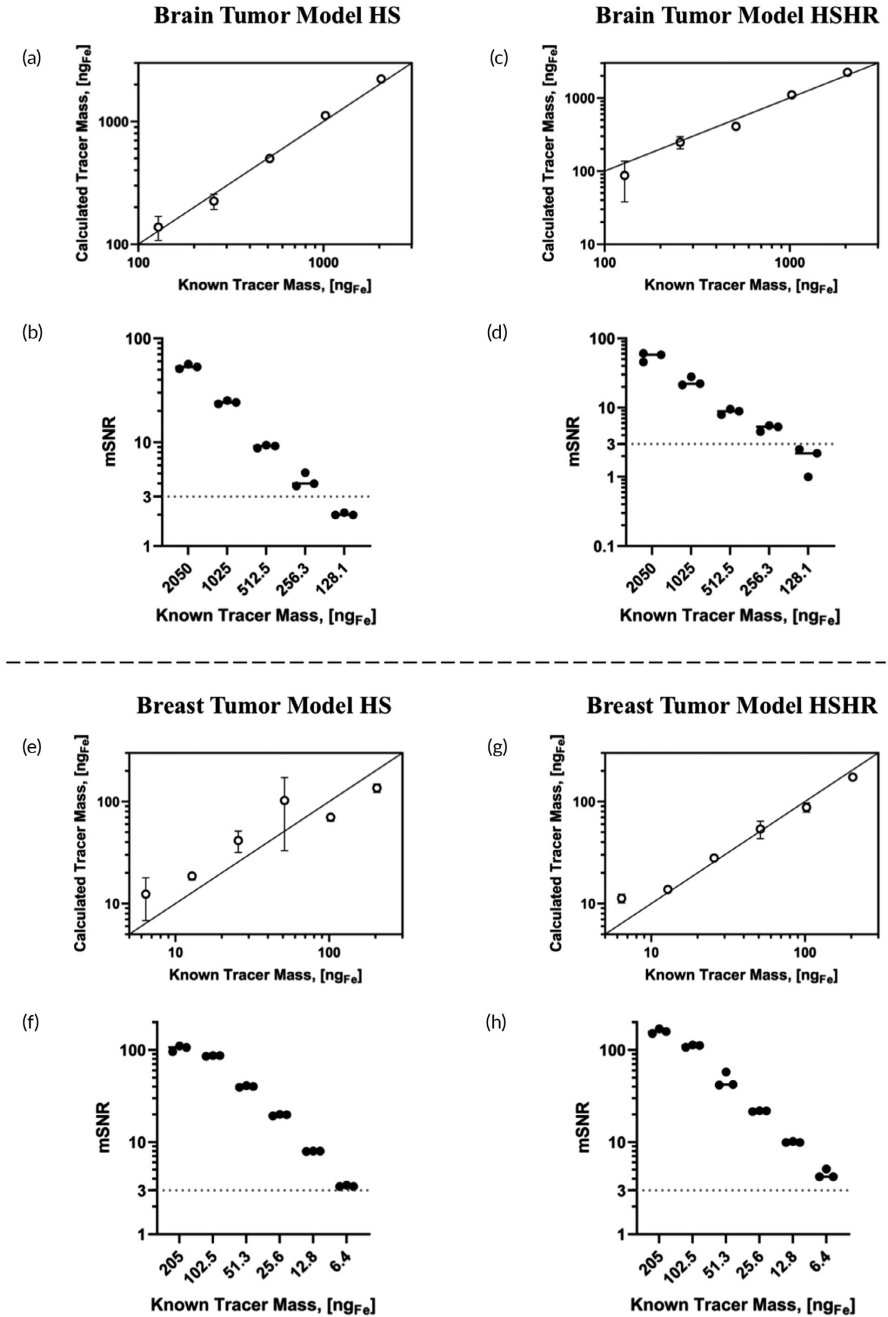
Quantitative analysis of the iron content in the tumor region of interest (ROI) for the brain (top) and breast (bottom) tumor models. Relationship between the known tracer mass and magnetic particle imaging (MPI) calculated tracer mass in the tumor ROI for the brain (a, c) and breast (e, g) tumor models in high‐sensitivity (HS) and high‐sensitivity/high‐resolution (HSHR) scanning modes. Mean signal to noise ratio (mSNR) plots of the tumor ROI for the brain (b, d) and breast (f, h) tumor models in HS and HSHR scanning modes. Some standard deviation bars (*n* = 3) are smaller than the markers and not clearly visible

Figure 4 summarizes the results of signal and iron quantification in the tumor ROI for the brain and breast tumor models for scans in HS and HSHR modes. The calculated iron mass in the ROI is compared to the known tracer mass loaded into the tumor phantom in panels (a) and (c) for the brain tumor model and panels (e) and (g) for the breast tumor model. The solid line corresponds to parity between the calculated and known tracer masses. In the brain tumor model, there seems to be very good agreement between the calculated and known tracer mass for all dilutions and both imaging modes. In contrast, for the breast tumor model there is better agreement between the calculated and known tracer mass for the HSHR scans, although the mass calculated from the HS scans retains the expected linear relation while overestimating the tracer mass. mSNR in the tumor ROIs were calculated and are shown in panels (b) and (d) for the brain tumor model and panels (f) and (h) for the breast tumor model. For the brain tumor model, dilutions 1–4 had mSNR above 3 in both imaging modes and dilution 5 had a mSNR slightly below 3. However, it is noted that dilution 5 had good agreement between calculated and known tracer masses, as shown in panels (a) and (b). For the breast tumor model, dilutions 1–6 had mSNR above 3 in both imaging modes, with dilution 6 being close to 3. Correspondingly, dilution 6 showed the greatest deviation from the parity line in panels (e) and (g) for the breast tumor model.

## 3 DISCUSSION

The 3D‐printed mouse phantoms presented in this study were created to evaluate the effect of MPI signal in the liver on quantification of tracer mass in locations corresponding to brain and breast tumors. Here, we have considered a signal to be above the LoD when the mSNR is greater than 3.^22^ Under these conditions, for the brain tumor model, the MPI signal was above the LoD for tumor regions with 256.2 ng_Fe_ in 2D (mSNR_HS_ = 4.3, mSNR_HSHR_ = 5.1) and 128.1 ng_Fe_ (mSNR_HS_ = 3.8), in the presence of ~10 μg_Fe_ tracer in the liver. For the breast tumor model, the MPI signal was above the LoD for tumor regions with 6.41 μg_Fe_ in 2D (mSNR_HS_ = 3.4, mSNR_HSHR_ = 4.5) and 1.60 μg_Fe_ in 3D (mSNR_HSHR_ = 6.4), in the presence of ~200 μg_Fe_ in the liver. These results demonstrate how a 3D‐printed mouse phantom can be used to evaluate the effect of nearby signals that may be present in in vivo studies and their effect on sensitivity and accuracy of quantification of tracer mass in a ROI. In this study, the iron content present in the liver was both chosen to mimic relevant studies that implement SPION administration. For the brain tumor model, an iron mass of ~10 μg_Fe_ was chosen based on a previous study that used MPI to monitor localization of SPION‐labeled T cell in the brain following ACT.^4^ In contrast, an iron mass of ~200 μg_Fe_ in the liver for the breast tumor model was chosen to mimic MFH studies, where a high presence of iron in the tumor site is required to achieve therapeutic temperatures. This causes a large quantity of iron to nonspecifically accumulate in the liver, which emphasizes the need to understand the effects of neighboring MPI signals on the tumor ROI. Due to the lower iron mass present in the liver of the brain tumor model, a lower detection limit was observed when compared to the breast tumor model. The HS and HSHR scan modes were both suitable for the brain tumor model and resulted in excellent agreement between MPI iron quantification and known tracer mass for a wide range of mSNR (Figure 4a–d). In contrast, the breast tumor model exhibited more accurate MPI quantification (Figure 4g) in the HSHR scan mode, likely due to better separation of the tumor and liver signal compared to the HS scan mode (Figure 4e). We note that a previous study^11^ reported a LoD of 28.3 ng_Fe_ for the RL‐1 SPION tracer used here when the tracer was evaluated without the interference of nearby signals, which is much lower than what was observed in this study. Our results demonstrate the importance of evaluating SPION tracer performance under anatomically correct conditions that mimic in vivo experiments to account for the effects of signal distribution from sites of tracer accumulation.

For image analysis, threshold ranges were chosen for each ROI segmentation based on a percentage of the maximum signal produced in the ROI. In the brain tumor model, a threshold range of 60% of the ROI's maximum MPI signal was used for all scanning modes. Analyses using different thresholding ranges for the brain tumor model in the HS scanning mode (Figure S3) suggest that this approach is accurate for a wide range of threshold values, whereas using a ROI defined by a brush resulted in an over estimation of the MPI quantification. In contrast, a thresholding range of 60% and 80% of the ROI's maximum MPI signal was used in the breast tumor model for HSHR and HS scan modes, respectively. The difference in thresholding for the breast tumor model is due to the high iron mass in the liver and its proximity to the tumor in the mammary region of the phantom, which caused a blooming artifact in the HS scan mode, causing the liver signal to bleed into other regions of the image, including the tumor ROI (Figure S4). The difference in gradient field strength of the MPI scan modes led to a more apparent signal blooming from the liver in the HS scan mode (3 T/m), compared to the HSHR scan mode (5.7 T/m). This blooming artifact is likely responsible for the decrease in accuracy and precision in MPI quantification for the HS scanning mode in the breast tumor model (Figure 4e). Furthermore, MPI overestimates the tracer mass in the breast tumor model at the lowest concentrations. This overestimation is most likely due to difficulty in selecting an ROI in 3D Slicer due to signal blooming from the liver. It is also important to note that because the lowest mass dilution that was visible in each model had a signal close to the background signal in the image, one can see imaging artifacts associated with background signal, especially around the liver. These image artifacts have different textures for images acquired using HS and HSHR modes due to differences in image reconstruction algorithms used by these two modes. Furthermore, these image artifacts do not affect signal quantification and analysis because they are due to the choice of color scale and because analysis is carried out with the full dynamic range of the signal in the data set.

The 3D‐printed mouse phantoms presented in this study can be used to optimize experimental planning by evaluating the effect of MPI scan modes on the ability to detect and differentiate tracer masses in anatomically correct locations. Thus, suitable SPION administration doses can be chosen based on the effect of nonspecific SPION accumulation in organs away from the target site. Since the 3D‐printed mouse phantom provides imaging data that mimic the mouse anatomy, appropriate image analysis approaches can be established, and image analysis algorithms can be developed and subsequently used to analyze data from in vivo studies. Importantly, MPI studies can utilize a 3D‐printed mouse phantom to optimize the planning and execution of in vivo studies before the use of animals. This greatly contributes to the advancement of 3Rs, which serve as the fundamental principles that underlie ethical and humane animal research.^16^ In terms of replacement, the 3D‐printed mouse phantom can answer important scientific questions regarding experimental design and analysis for MPI studies without the use of animals. The controlled model of the mouse phantom can ensure experiments and analyses are robust and can prevent the need for additional animal studies, contributing to reduction. For refinement, the MPI is a novel and noninvasive imaging modality which advances the welfare of animals and can minimize the number of animals sacrificed for longitudinal studies.

While this study presents the use of the 3D‐printed mouse phantom for a brain and breast cancer model, this phantom is extremely versatile and can be modified using computer aided design (CAD) software to include or exclude selected organs to simulate various disease models. In this study, the surfaces of the Digimouse, a tessellated 3D anatomical mouse atlas presented in Dogdas et al., were converted to stereolithography (STL) files and modified to create representative phantoms for a breast and brain tumor model.^17^ The major components of the phantoms were the liver and the brain and mammary fat pad tumors. This allowed evaluation of the effect of nonspecific accumulation of SPION tracer in the liver on signal detection and quantification in ROI corresponding to brain and breast tumors. However, the Digimouse atlas contains 3D segmentations of several organs in the normal nude male mouse, such as the heart, lungs, stomach, spleen, kidneys, and bladder, to name a few.^17^ This allows for creation of anatomically correct phantoms representative of numerous disease models for a variety of applications. Alternative digital mouse atlases have also been developed to model particular sets of organs and mice at different developmental stages.^18^, ^19^, ^20^, ^21^ These atlas data sets could be combined and utilized to create anatomically correct phantoms to further tailor these tools to specific applications.

A limitation of this study was that SPION tracer mass in the liver was held constant for each tumor model. Additional work would be needed to correlate liver tracer mass with corresponding sensitivity. Moreover, the static 3D‐printed mouse phantom does not allow for dynamic processes such as breathing or fluid flow, meaning signal due to tracer circulating in the blood cannot be accounted for. As such, these 3D‐printed mouse phantoms are suitable to assess situations where MPI scans are acquired after the tracer has cleared from the blood. Previous MPI studies have developed phantoms that enable fluid flow^9^, ^13^ and the associated methods could be applied to the anatomically correct mouse phantom reported here in future studies. An additional limitation of the 3D‐printed mouse phantom reported here involves the resolution of 3D printers, as it is challenging to print structures smaller than 1 mm. Currently, the resolution of MPI is greater than or equal to 1 mm,^23^ alleviating the need for 3D‐printed structures smaller than this. However, as new MPI tracers optimized for high resolution are developed there will be a need for alternative approaches that allow for printing phantoms with greater than 1 mm resolution. Despite these limitations, this study demonstrates the necessity of 3D‐printed phantoms that simulate in vivo experiments to optimize experimental planning and analysis, prior to the use of animals.

## 4 MATERIALS AND METHODS

### 4.1 3D‐printed mouse phantom creation

CAD techniques were used to prepare 3D‐printable models of mouse anatomy from surface tessellation data. For this purpose, we used the Digimouse 3D whole body mouse atlas reported by Dogdas et al. and which was generated using a combination of CT, PET, x‐ray, and cryosection techniques.^17^ Surface meshes for the mouse body, liver, and brain were isolated using the Digimouse anatomical tessellation data.^17^ Details of the methodology for converting surface tessellations to a 3D‐printed model are outlined below. In brief, meshes were reduced and patched, converted to solid parts, edited, and 3D printed as an assembly of a whole mouse body with hollow organs to be loaded with SPION tracer.

**FIGURE 5:**
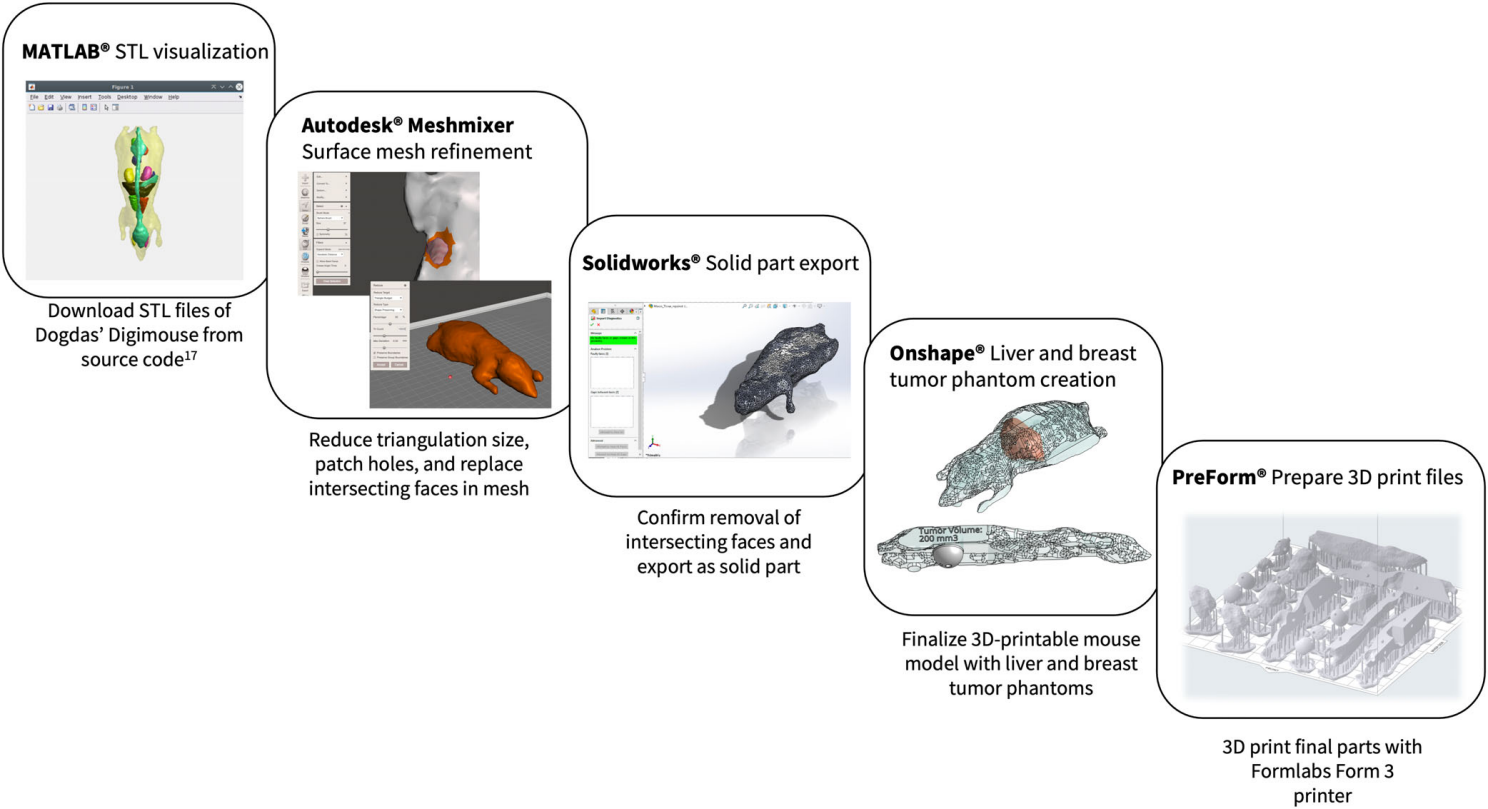
Flowchart demonstrating the conversion of the Digimouse tessellation data into modifiable and 3D‐printable imaging phantoms

Tessellations of mouse anatomy were generated by running the Digimouse Tessellated_Atlas.mat visualization script in MATLAB® (MathWorks). This Digimouse visualization and volume tessellation code is publicly available and can be found at https://neuroimage.usc.edu/neuro/Digimouse.^17^ Meshes were saved as STL files and imported into Autodesk® MeshMixer (Autodesk Inc) to reduce their triangle number, patch holes, and replace intersecting faces in the mesh. Using SolidWorks® (Dassault Systèmes SolidWorks Corporation), these refined mouse body and liver meshes were saved as solid part STL files and subsequently imported into Onshape® (Onshape Inc) and Blender® (Blender Foundation) for final design modifications before printing. Figure 5 outlines this conversion process.

The mouse liver was made hollow in Blender® using a 0.8 mm minimum wall thickness and holes were added to the liver for direct SPION loading. Two holes were added through the mouse brain and suited to hold a capillary tube (1/32″ ID × 1/16″ OD) loaded with SPIONs in a vertical or horizontal position. For this study, SPION loaded capillary tubes were placed in the horizontal position, coincident with the coronal plane. The mouse body, liver, and brain were then imported into Onshape® and Boolean relationships were used to create a body cavity for each organ. The body was then segmented into a four‐piece assembly using sagittal and transverse plane slices to easily insert and remove the organs. In a separate model, a ventral right flank cavity was designed to fit interchangeable hollow spherical breast tumors of various volumes ranging from 10 to 1000 mm^3^ for SPION loading and subsequent MPI analysis. The mouse phantom was printed with the Form 3 STL printer (Formlabs Inc) using Clear V4 resin (Formlabs Inc) with layer sizes ranging from 25 to 100 μm.

### 4.2 Nanoparticle synthesis

RL‐1 is a SPION tracer with magnetic properties that have been optimized for MPI and was prepared by the methods described by Liu et al.^11^ Briefly, these MPI‐tailored SPIONs were synthesized by thermal decomposition of iron oleate with the addition of molecular oxygen and coated with covalently bonded polyethylene glycol. In this study, the batch RL‐1C was used, which had a core diameter of ~21.4 ± 2.4 nm and hydrodynamic diameter of ~55 ± 20 nm.^11^

### 4.3 Sample preparation

A serial dilution of RL‐1 particles, with dilution factor of 2, was prepared with concentrations ranging from 2050 to 8 μg_Fe_/mL. Iron content was calculated by the 1,10‐phenanthroline colorimetric assay for iron quantification and all samples were prepared as a 1:1 mixture of RL‐1 and Omnipaque, a CT contrast agent. For the brain and breast tumor models, the liver cavity was loaded with a total iron mass of 10 and 200 μg_Fe_, respectively, in approximately 375 μL. For both models, the iron content present at the tumor site was varied using the RL‐1 dilution series. For the brain tumor model, a capillary tube was loaded with a constant volume of 1 μL. The breast tumor model contained a 3D‐printed 100 μL volume tumor in the mammary fat pad location and was loaded with the dilution series. Three fiducial samples were prepared and loaded into capillary tubes (1/32″ ID × 1/16″ OD) with a constant volume of 1 μL. For the brain tumor model, two fiducials featured a total iron mass of 1 μg_Fe_ and the third fiducial contained 0.5 μg_Fe_. All three fiducials contained a total iron mass of 2 μg_Fe_ for the breast cancer model. Table 1 summarizes the total iron mass featured in the tumor and liver for both models.

### 4.4 Data acquisition

2D and 3D MPI scans were obtained on the MOMENTUM™ preclinical scanner (Magnetic Insight) using both HS (3.055 T/m gradient strength) and HSHR (5.7 T/m gradient strength) scanning modes. Isopropanol was used to clean the MPI beds and blank scans were acquired to assess signal contamination prior to sample imaging. The particle‐loaded mouse phantom was placed in a 3D‐printed MPI holder bed in a head‐first prone and head‐first supine orientation for the brain and breast tumor models, respectively. Optical images of phantom placement on the FOV (6 × 12 cm) were taken on the MOMENTUM™ prior to each scan. 2D scans were performed in triplicate (*n* = 3) by replacing the tumor with another of the same iron mass, while keeping the same liver and fiducials for each dilution. 3D MPI scans were obtained for one trial (*n* = 1) for each dilution with both models. The time for MPI acquisition was ~3 min for each 2D scan and ~42 min for each 3D scan. CT scans were obtained on an IVIS® SpectrumCT from PerkinElmer following each HS and HSHR 3D MPI scan.

### 4.5 Data analysis

All MPI scans were analyzed using 3D Slicer (Slicer 4.10.2), an open‐source software for medical imaging analysis.^24^ Image registration was performed with the landmark registration tool to colocalize fiducials on 2D and 3D MPI scans with optical images and CT scans, respectively. Since all fiducials contained Omnipaque, a CT contrast agent, the point source of the fiducial could be visualized and located in the CT scans to properly align with the MPI data. The fiducial placement was kept constant throughout the study. For analysis of MPI scans, the thresholding tool in 3D Slicer was used to create segmentations for each ROI, corresponding to fiducial 1, fiducial 2, fiducial 3, tumor, liver, and background (Figure S5). Unique thresholding ranges were chosen for each segmentation based on a percentage of the maximum intensity signal in the ROI. For the brain tumor model, thresholding ranges were based on 60% of the ROI's maximum signal for both HS and HSHR scan modes and for all segmentations excluding the background. For the breast cancer model, HS and HSHR scans were analyzed with a threshold value of 80% and 60% from the maximum MPI signal in each ROI, respectively. The background segmentation for both models used thresholding values that ranged from the minimum MPI signal intensity present in the image to three times the standard deviation of a blank MPI scan (3 × SD_blank_) in the corresponding scan mode.

To compare analysis methods, an additional technique using the “Paint” function in 3D Slicer was used to create a defined circle ROI with a 15 mm brush diameter for the brain tumor ROI in the 2D HS scanning mode. The brush diameter was chosen to encompass the MPI signal produced from the brain ROI in the first dilution and used for all subsequent dilutions.

The LoD was evaluated based on the calculation of the mean signal to noise ratio (mSNR) of the tumor ROI. The mSNR was calculated as the ratio of the mean MPI signal intensity of the tumor ROI to the standard deviation of the background ROI in the same scan. The LoD was determined to be the smallest tumor iron mass with a mSNR > 3. The MPI iron quantification was calculated by considering the background signal and the known iron mass present in the fiducials. First, the total signal in the ROI was calculated using Equation (1). Equation (2) was used to calculate the contribution of the background signal to the sample ROI signal by multiplying the mean signal present in the background ROI and the voxels of the sample ROI. To eliminate the background signal contribution, Equation (3) was used to subtract the effects of the background signal that may influence the ROI sample signal. Then, Equation (4) was used to determine the ratio between the known iron mass present in a fiducial and the signal produced by the ROI sample with the background signal subtracted. Finally, the MPI mass was calculated using Equation (5), which multiplies the background subtracted ROI signal and the average mass per signal ratio.

(1) $${\text{Total Signal}}_{\mathrm{ROI}}=\#{\text{Voxels}}_{\mathrm{ROI}}*{\text{Mean}}_{\mathrm{ROI}}$$

(2) $$\mathrm{Bkg}\;{\text{Signal}}_{\mathrm{ROI}}=\#{\text{Voxels}}_{\mathrm{ROI}}*{\text{Mean}}_{\mathrm{Bkg}}$$

(3) $$\mathrm{Bkg}\;\text{Subtracted Signal}={\text{Total Signal}}_{\mathrm{ROI}}-\mathrm{Bkg}\;{\text{Signal}}_{\mathrm{ROI}}$$

(4) $$\frac{\text{Mass}}{\text{Signal}}=\frac{\text{Known}\;{\text{Iron Mass}}_{\text{Fiducial}}}{\mathrm{Bkg}\;\text{Subtracted Signal}}$$

(5) $$\mathrm{MPI}\;\text{Mass}=\mathrm{Bkg}\;\text{Subtracted Signal}*\bar{\frac{\text{Mass}}{\text{Signal}}}$$

## 5 CONCLUSIONS

The development of molecular imaging phantoms that mimic in vivo experimental conditions greatly aids in the optimization of experimental design for MPI studies. The anatomically correct 3D‐printed mouse phantoms reported here allow for in vivo experiment planning and validation of MPI quantification workflows without the need for animal experimentation. The utility of the 3D‐printed mouse phantom was illustrated with phantoms representative of brain and breast tumor animal models. The accuracy of an approach to quantify tracer mass in ROI was demonstrated based on comparison of calculated tracer mass to known tracer mass in the brain and breast tumor cavities, while a constant tracer mass resided in the liver cavity. The reported experiments also demonstrate that the LoD in MPI is sensitive to the presence of tracer in nearby locations, such as in the liver. It was found that signal from the liver cavity in the phantoms affected the LoD, with greater tracer mass in the liver cavity resulting in poorer sensitivity in tumor regions. The results presented here suggest MPI experimental parameters can be successfully iterated and analyzed for a brain and breast tumor model to evaluate impact of liver accumulation on MPI sensitivity and to assess the accuracy of MPI quantification methods. The capabilities of this 3D‐printed anatomically correct mouse phantom are extensive, as it is possible to modify the phantom using CAD software to simulate a variety of disease models.

## AUTHOR CONTRIBUTIONS

**Nicole S. Sarna:** Conceptualization (equal); data curation (equal); formal analysis (equal); investigation (equal); methodology (equal); visualization (equal); writing – review and editing (equal). **Leyda Marrero‐Morales:** Conceptualization (equal); data curation (equal); formal analysis (equal); investigation (equal); methodology (equal); visualization (equal); writing – review and editing (equal). **Ryan DeGroff:** Conceptualization (equal); methodology (equal); resources (equal); writing – review and editing (supporting). **Angelie Rivera‐Rodriguez:** Conceptualization (supporting); methodology (supporting); supervision (supporting); validation (supporting); writing – review and editing (supporting). **Sitong Liu:** Resources (equal); writing – review and editing (supporting). **Andreina Chiu Lam:** Resources (equal); writing – review and editing (supporting). **Hayden J. Good:** Resources (equal); writing – review and editing (supporting). **Carlos M. Rinaldi‐Ramos:** Conceptualization (lead); formal analysis (supporting); methodology (lead); project administration (lead); supervision (lead); writing – review and editing (supporting).

## CONFLICT OF INTEREST

The authors have no conflicts of interest to declare.

### PEER REVIEW

The peer review history for this article is available at https://publons.com/publon/10.1002/btm2.10299.

## Supporting information


**Figure S1** Montage of images for dilution series in HS mode in the brain tumor model. Fiducial 1, 2, and 3 contain an iron mass of 0.5, 1, and 1 μg_Fe_, respectively, and the liver contains 10.3 μg_Fe_. The color lookup table range was kept the same for all MPI scans and was selected to best visualize the breast tumor signal at low concentrations.

**Figure S2** Montage of images for dilution series in HS mode in the breast tumor model. Fiducial 1, 2, and 3 contain an iron mass of 2, 2, and 1 μg_Fe_, respectively, and the liver contains 205 μg_Fe_. The color lookup table range was kept the same for all MPI scans and was selected to best visualize the breast tumor signal at low concentrations, which may make it difficult to visualize fiducial MPI signal.

**Figure S3** Comparison of signal quantification for brain tumor HS mode images at different threshold levels and comparison to using an ROI defined using a brush with a diameter of 15 mm in each image.

**Figure S4** Representative images of the breast tumor model in HS (A) and HSHR (B) scanning mode. Blooming artifact from the liver is more apparent in the HS scan mode when compared to the HSHR scan mode, which caused difficulties in accurately defining the tumor ROI.

**Figure S5** Representative MPI scan of the brain tumor model with overlaid ROI's using unique thresholding ranges based on the maximum signal of each ROI. Fiducial 1, 2, and 3 contain an iron mass of 0.5, 1, and 1 μg_Fe_, respectively, the brain contains 2.05 μg_Fe_, and the liver contains 10.3 μg_Fe._

**Movie S1** 3D visualization of the brain tumor model. 3D MPI scan co‐registered with CT scan with 2.05 μg_Fe_ present in the brain tumor and approximately 10 μg_Fe_ in the liver. MPI signal produced from the fiducials may not be visible due to the selected color lookup table range.

**Movie S2** 3D visualization of the breast tumor model. 3D MPI scan co‐registered with CT scan with 102.5 μg_Fe_ present in the breast tumor and approximately 200 μg_Fe_ present in the liver. MPI signal produced from the fiducials may not be visible due to the selected color lookup table range.

## Data Availability

The data that support the findings of this study are available from the corresponding author upon reasonable request.
