# Supplementary figures and images for: An anatomically correct 3D‐printed mouse phantom for magnetic particle imaging studies
Source: Bioeng Transl Med. 2022 Mar 1;7(3):e10299. doi: 10.1002/btm2.10299 (PMC9472006; doi:10.1002/btm2.10299)

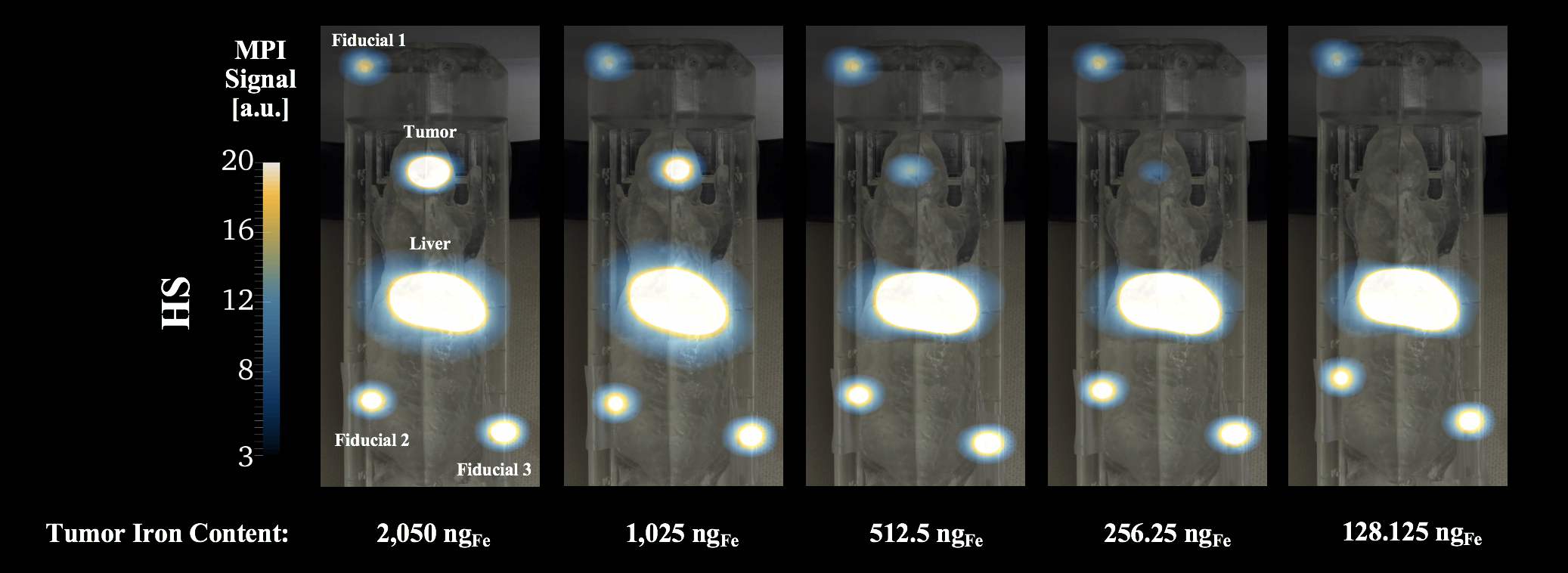

Supplement: Supplementary file 1 — Figure S1 Montage of images for dilution series in HS mode in the brain tumor model. Fiducial 1, 2, and 3 contain an iron mass of 0.5, 1, and 1 μgFe, respectively, and the liver contains 10.3 μgFe. The color lookup table range was kept the same for all MPI scans and was selected to best visualize the breast tumor signal at low concentrations. [file BTM2-7-e10299-s002.png]

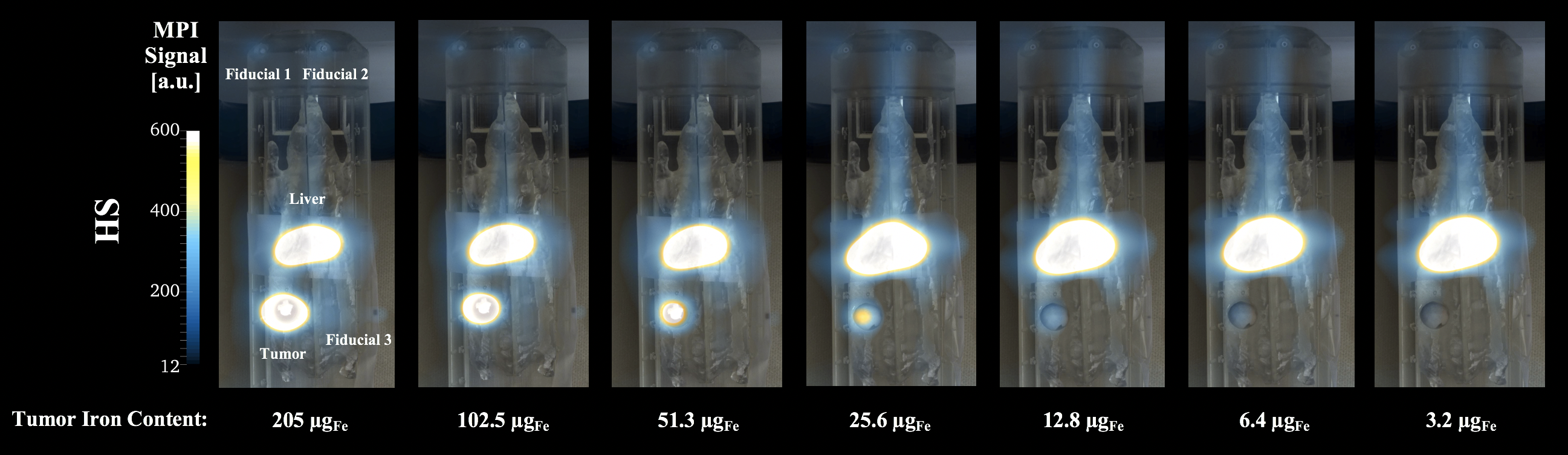

Supplement: Supplementary file 2 — Figure S2 Montage of images for dilution series in HS mode in the breast tumor model. Fiducial 1, 2, and 3 contain an iron mass of 2, 2, and 1 μgFe, respectively, and the liver contains 205 μgFe. The color lookup table range was kept the same for all MPI scans and was selected to best visualize the breast tumor signal at low concentrations, which may make it difficult to visualize fiducial MPI signal. [file BTM2-7-e10299-s001.png]

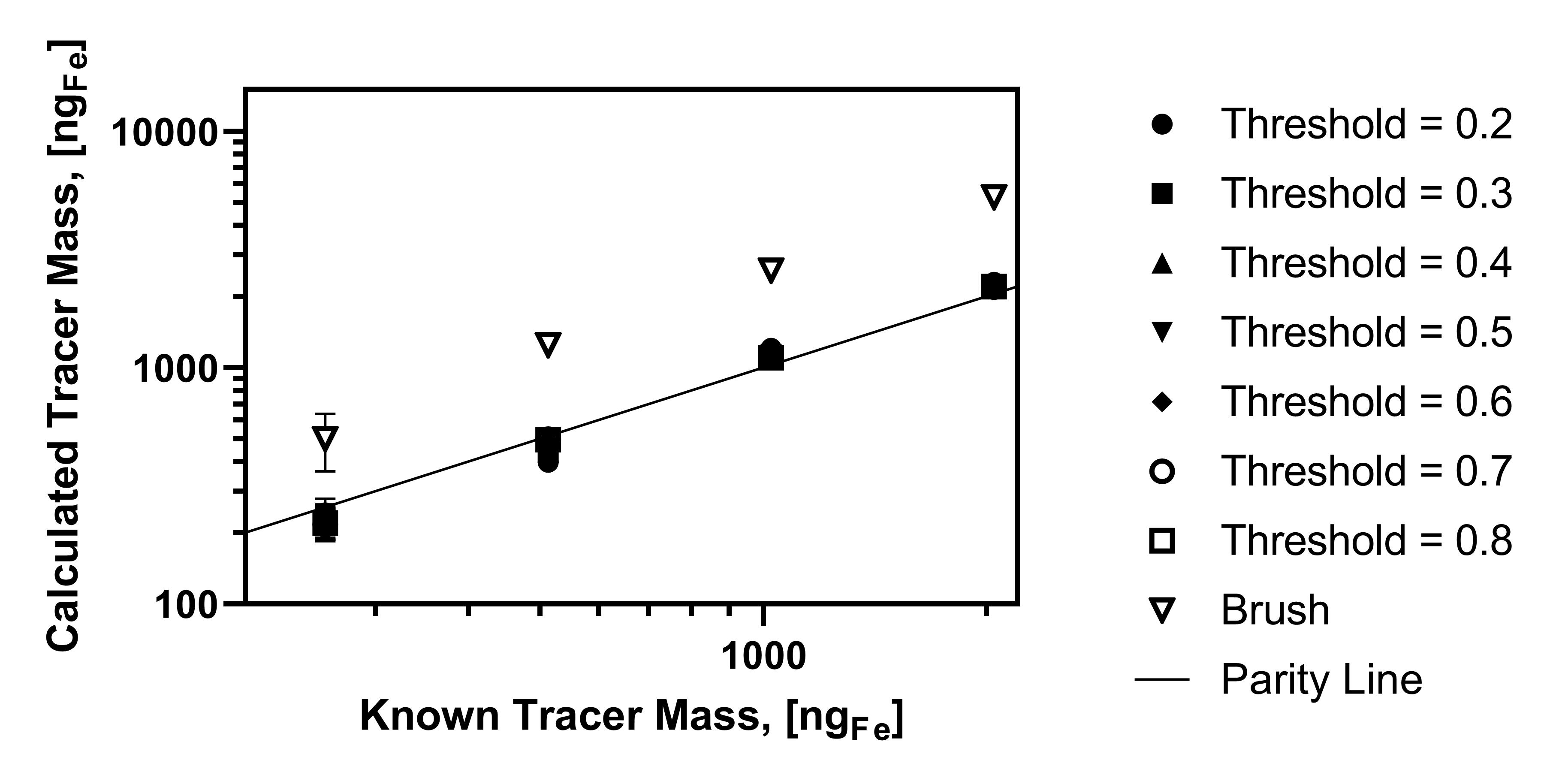

Supplement: Supplementary file 3 — Figure S3 Comparison of signal quantification for brain tumor HS mode images at different threshold levels and comparison to using an ROI defined using a brush with a diameter of 15 mm in each image. [file BTM2-7-e10299-s004.png]

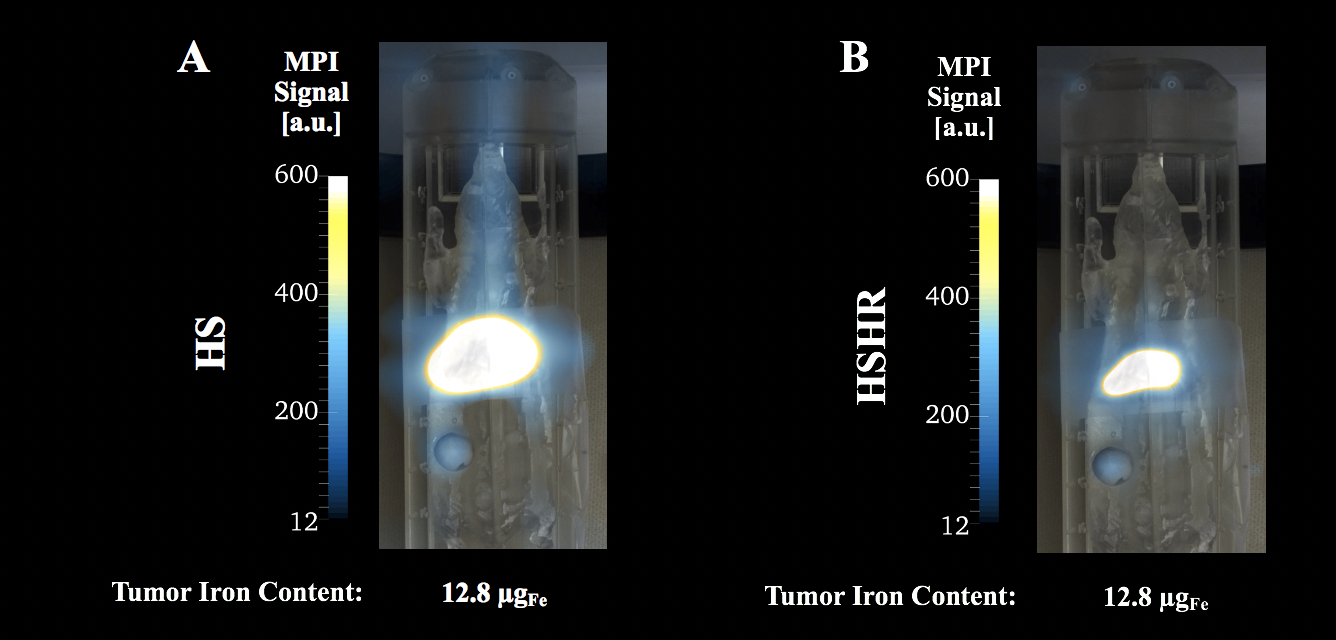

Supplement: Supplementary file 4 — Figure S4 Representative images of the breast tumor model in HS (A) and HSHR (B) scanning mode. Blooming artifact from the liver is more apparent in the HS scan mode when compared to the HSHR scan mode, which caused difficulties in accurately defining the tumor ROI. [file BTM2-7-e10299-s007.png]

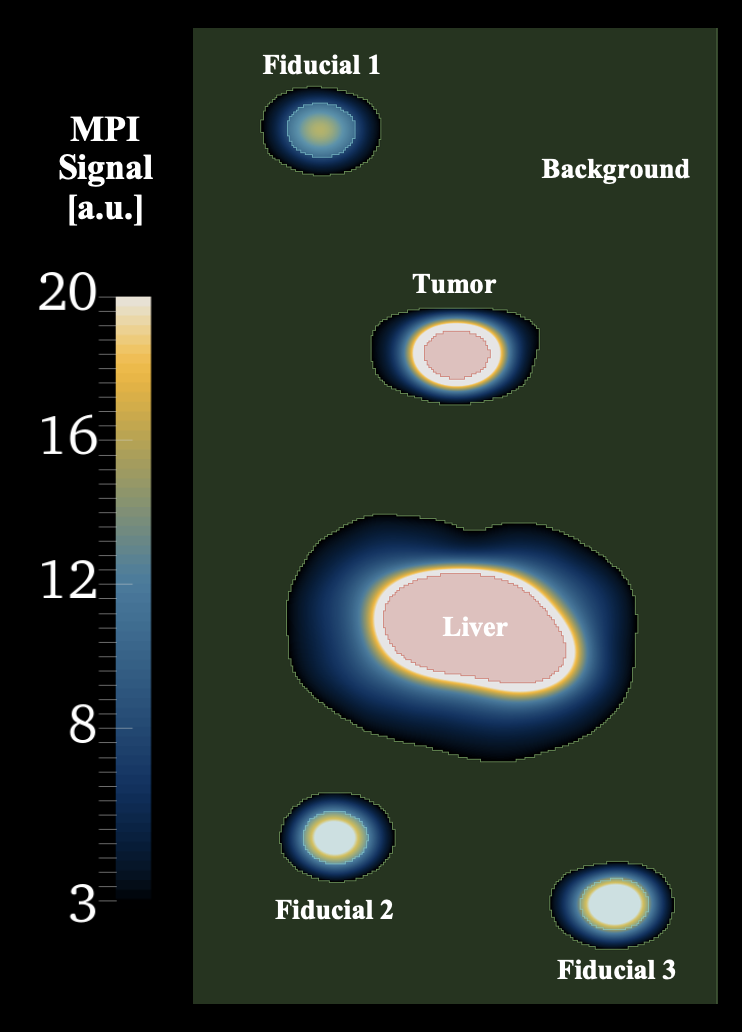

Supplement: Supplementary file 5 — Figure S5 Representative MPI scan of the brain tumor model with overlaid ROI's using unique thresholding ranges based on the maximum signal of each ROI. Fiducial 1, 2, and 3 contain an iron mass of 0.5, 1, and 1 μgFe, respectively, the brain contains 2.05 μgFe, and the liver contains 10.3 μgFe. [file BTM2-7-e10299-s005.png]
